# Improved brain MRI indices in the acute brain stem infarct sites treated with hydroxyl radical scavengers, Edaravone and hydrogen, as compared to Edaravone alone. A non-controlled study

**DOI:** 10.1186/2045-9912-1-12

**Published:** 2011-06-07

**Authors:** Hirohisa Ono, Yoji Nishijima, Naoto Adachi, Shigekuni Tachibana, Shiroh Chitoku, Shigeo Mukaihara, Masaki Sakamoto, Yohei Kudo, Jun Nakazawa, Kumi Kaneko, Hiroshi Nawashiro

**Affiliations:** 1 Department of Neurosurgery, Nishijima Hospital, Oooka, Numazu City, Sizuoka, 410-0022, Japan; 2 Department of Neurosurgery, National Defence Medical College, Tokorozawa City, Saitama,359-8513, Japan

## Abstract

**Background:**

In acute stage of cerebral infarction, MRI indices (rDWI & rADC) deteriorate during the first 3-7 days after the ictus and then gradually normalize in approximately 10 days (pseudonormalization time), although the tissue is already infarcted. Since effective treatments improve these indices significantly and in less than the natural pseudonormalization time, a combined analysis of these changes provides an opportunity for objective evaluation on the effectiveness of various treatments for cerebral infarction. Hydroxyl radicals are highly destructive to the tissue and aggravate cerebral infarction. We treated brainstem infarction patients in acute stage with hydroxyl radical scavengers (Edaravone and hydrogen) by intravenous administration and evaluated the effects of the treatment by a serial observation and analysis of these MRI indices. The effects of the treatment were evaluated and compared in two groups, an Edaravone alone group and a combined group with Edaravone and hydrogen, in order to assess beneficial effects of addition of hydrogen.

**Methods:**

The patients were divided in Edaravone only group (E group. 26 patients) and combined treatment group with Edaravone and hydrogen enriched saline (EH group. 8 patients). The extent of the initial hump of rDWI, the initial dip of rADC and pseudo-normalization time were determined in each patient serially and averages of these data were compared in these two groups and also with the natural course in the literatures.

**Results:**

The initial hump of rDWI reached 2.0 in the E group which was better than 2.5 of the natural course but was not as good as 1.5 of the EH group. The initial dip of rADC was 0.6 in the E group which was close to the natural course but worse than 0.8 of the EH group. Pseudonormalization time of rDWI and rADC was 9 days only in EH group but longer in other groups. Addition of hydrogen caused no side effects.

**Conclusions:**

Administration of hydroxyl radical scavengers in acute stage of brainstem infarction improved MRI indices against the natural course. The effects were more obvious and significant in the EH group. These findings may imply the need for more frequent daily administration of hydroxyl scavenger, or possible additional hydrogen effects on scavenger mechanisms.

## Background

Clinical care of cerebral infarction patients begins with visual evaluation of MRI (magnetic resonance image). It is well known now that the diffusion based MRI sequences can detect the abnormality within minutes after the onset of severe ischemia in the brain tissue. However, the differences in the MRI scan machinery, display software and filing methods may make the visual interpretation of the MRI images sometimes inconsistent. The diffusion data are more useful when presented as a comparison to those in the identical area of the other side of the brain, because in this way, all the hardware related inconsistency can be removed. The comparison utilizes a ratio of the MRI data, particularly the data capable of determining the degree of water molecule diffusion in the tissue such as DWI (Diffusion Weighted Image) and ADC (Apparent Diffusion Coefficient). The ratio is calculated by dividing the data in the pathological side by those in the normal side and designated as rDWI (relative DWI) and rADC (relative ADC).

The cells in severely ischemic brain tissue swell due to accumulation of water and electrolytes in the cells, immediately after the Na pump fails. The swelling reduces the extracellular space where the free motion of water molecules was a major source of the tissue diffusion. Thus, MRI indices (rADC and rDWI) deteriorate within minutes after the Na pump failure and continue to get worse for the first 3 to 5 days in the infarcted brain tissue [1], unless recanalization or restoration of blood flow occurs [2]. The deterioration of the indices is characterized by the initial rDWI increase (initial hump) up to 2.5 or higher and the initial rADC decrease (initial dip) down to 0.6 or below [3], reaching to a lowest value on Day3 [4]. Then, both indices gradually return to close to a normal level or 1.0, despite of the fact that the tissue is already infarcted (pseudonormalization) in 10 to 11 days (pseudo normalization time) after the ischemic ictus in the white matter [4]. After the pseudonormalization, rADC continues to increase (late hike) for many months [5,6]. However, recanalization treatment alters this natural course dramatically and the hump and the dip of diffusion related MRI indices may not appear at all and the pseudonormalization time shortens significantly down to 24 hrs or less after the treatment [7,8], only when the recanalization successfully restores the blood flow in the area. Although recanalization treatment such as with tPA (tissue plasminogen activator) is the most potent treatment of all for acute cerebral infarction, the treatment needs to be started within 3 hrs after the onset of the symptoms and has to satisfy rigid criteria. Therefore, except for few lucky tPA treated patients, the majority of the acute cerebral infarct patients are currently treated with diverse medications, including scavengers of reactive oxygen species (ROS). The ROS aggravate the ischemic tissue by a self-propagating chain reaction of depriving another electron from near-by molecules. In Japan, Edaravone (3-methyl-1-phenyl-2-pyrazolin-5-one) [9] is the only medication approved since 2001 for the use in acute stage of cerebral infarction patients as a scavenger of hydroxyl radicals and a neuroprotectant [10].

However, in our preliminary study, the treatment of acute cerebral infarction with Edaravone improved the initial hump and the initial dip of the MRI indices only slightly and it shortened the pseudo normalization time but rather mildly. Edaravone is known to have a rather short t1/2 beta, or elimination half life of the drug level, particularly in elderly patients who occupy a majority of cerebral infarction population. In addition, Cmax, or maximum drug concentration in the blood, of the Edaravone, with currently approved intravenous administration of 30 mg remains at about 1/10 level of a standard 1-10 micromole concentration used in many in vitro experiments. In addition, because of possible side effects, Edaravone may not be given to the patients who have compromised liver or kidney function and also not more than twice a day according to the governmental approval. On the other hand, molecular hydrogen, which is well known to have potent scavenger actions against hydroxyl radicals and related harmful oxidation [11] had no risk of complications in our preliminary study even on the patients who had already established kidney or liver disease. Our current study was designed to supplement possible low and short blood level of Edaravone with hydrogen for the treatment of acute cerebral infarction. The effects of the supplementing with hydrogen were evaluated by comparing the results of the treatment in a group treated with Edaravone only (E group) and in a combined Edaravone and hydrogen group (EH group) and also against the natural course published in the literatures [1-6]. Since subtle neurological changes after cerebral infarction during the acute stage are sometimes difficult to substantiate, a totally objective method using MRI indices, rADC and rDWI, was adopted for the evaluation. These indices were calculated at the infarction sites of the patients serially and averaged and compared daily in the two groups. In addition, regular neurological evaluation of the patients was done mainly with NIHSS (NIH stroke score).

## Methods

### Patients

Consecutive 34 patients who were diagnosed as having cerebral infarction of BAD type (branch atheromatous disease) in the brainstem were enrolled in the study. All of these patients lived in the local area of our hospital and were brought in within 4 to 24 hours after the onset of the symptoms. The first 26 patients were treated with Edaravone alone (E group) and the following 8 patients received hydrogen-rich intravenous fluid in addition to Edaravone (EH group). For the EH group of 8 patients, intravenous Edaravone (30 mg Edaravone Kit) was given at 6 AM and 6 PM as a regular schedule and hydrogen-rich intravenous solutions were added at 10 AM and 4 PM. These treatments lasted for 7 days. Neurological status was recorded essentially with NIHSS and compared at the time of admission and discharge from the hospital. The neurological evaluation was based upon the NIHSS method and was equally performed in the two groups. Since the dramatic and substantial improvements in clinical conditions and MRI indices after recanalization may overwhelm any effects of other medications, only those patients who were diagnosed as stroke due to branch atheromatous disease (BAD), which is a non-recanalization type cerebral infarction, in the brainstem were recruited. BAD involves perforating arteries particularly at lateral striate artery (LSA) region or at parapontine artery (PPA) region and is known as a type of progressive stroke [12] also.

The informed consent in a form approved by the Nishijima Hospital Ethics Committee was obtained from all the patients before the treatment or from their legal guardians when the patients could not sign the consent, by the time of initiation of the treatment.

### Production of hydrogen-rich intravenous fluid

Regular intravenous fluid bags were immersed, without opening the bag and without adding any alteration on the bag, in a hydrogen water tank which is capable of producing hydrogen-rich water up to 1.6 ppm concentration (Miz.Co, Fujisawa, Japan, Patent No.4486157, Patent Gazette of Japan 2010). The hydrogen concentration increased in the bag by diffusion through the totally intact wall of the plastic bag to more than 250 micromole/L and to saturation, depending upon the duration of the immersion and temperature. A saline bag of 250 ml size (Terumo Co. Tokyo, Japan) and a maltose solution bag of 200 ml size (Airomu Co. Atsugi, Japan) were chosen according to the highest diffusibility of the bag wall we could find.

### MRI analysis

MRI signal intensities in DWI and ADC of each infarction site were observed first and then, serial changes of these images were compared in the E group and the EH group. The DWI and ADC signal intensities were also compared with those in the exactly same area of the other side of the brain and the ratio was calculated as rDWI (relative DWI) and rADC (relative ADC). Averages of these indices were compared in the two groups and also with the previous publications by using the data in the literature [3] for a statistical significance. A special attention was paid for the determination of abnormal area. Firstly, all of the MRI images of the patient were reviewed and the largest area of the abnormality was chosen to be the site and size of the lesion for the calculation and the pixel size of the area were recorded. Then, the area was copied on a transparent film together with surrounding recognizable structures as a template, which was used for calculation of the remainder of MRIs. This is to prepare, in case of size changes of the abnormality or even disappearance of the abnormality, to calculate the indices exactly in the same area and in a same manner. If an ADC map was not distinct enough by the naked eye, then the DWI template was used to define the area of abnormality. The MRI scan was taken on the day of admission (Day1) and follow-up MRIs were scheduled to be taken every other day but this could not be accomplished in every patient when other tests such as patient's vascular evaluation or cardiopulmonary function test were thought to be more urgent.

The study was approved by Nishijima Hospital Ethics Committee and the production of hydrogen rich IV fluid as "Hospital Preparation" and its clinical use in Nishijima Hospital, were conducted upon the advice from Nishijima Hospital Pharmacists Council and Japanese Welfare-Labour Administration (Tokai-Hokuriku District Bureau) and Sizuoka Prefectural Administration (Pharmaceutical Affair, Regulatory Audit Section).

## Results

### MRI images (DWI and ADC) of infarction areas and comparison of the images in the E group (treated with Edaravone only, Figure 1 upper) and the EH group (treated with a combination of Edaravone and hydrogen, Figure 1 lower)

**Figure 1:**
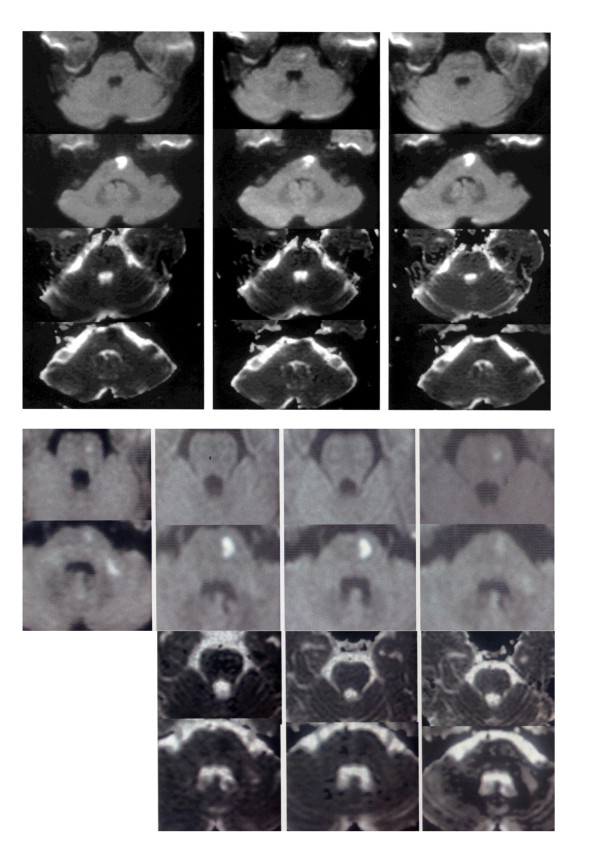
**Serial MRI changes in the upper brain stem lesion slices (1st & 3rd row) and lower brain stem lesion slices (2nd and 4th row) of DWI (1st & 2nd row) and ADC (3rd & 4^th ^row) images**. **upper. Serial MRI of a representative patient in E group on Day 1, 3, 6 (left to right)**. The lesion involved two adjacent slices at the upper (1st row) and lower (2nd row) brain stem. The DWI signal intensity (whiteness) of the upper slice increased on Day3 (presence of the initial hump), but remained almost unchanged on Day 1,3& 6 in the lower slice (2nd row) by the naked eye. The reduced ADC signal intensity (blackness) of the same lesion was seen even on Day6, particularly in the lower lesion slice (4th row). **lower. Serial MRI of a representative patient in EH group on Day1, 2, 7, 9 (left to right)**. The lesions also involved two adjacent slices. The DWI signal intensity of the upper slice (1st row) was seen on Day1 but was invisible on the Day2 &7 (absence of the initial hump). The initial hump was seen only in the anterior part of the lower lesion slice (2nd row) but not in the posterior-lateral extension of the lesion towards the cerebellum which had disappeared on Day2 & 7(absence of the initial hump). The ADC signal was clearly darker in the lower brainstem lesion (4th row) on Day 2 but disappearing on Day7 and became grey colour on Day9 (shortened pseudonormalization time and late hike, 4th row, right end).

The results were firstly evaluated by MRI images (DWI and ADC) without indices (Figure 1). The DWI images generally showed increased signal intensity (appeared with more whiteness) at the infarction sites in both groups. The ADC images, on the other hand, showed decreased signal intensity (appeared with more blackness) at the lesion sites, which were rather difficult to see as compared to the lesions in DWI images. These signal intensities of the lesions in the E group and the EH group differed obviously in many cases but in some cases, the differences were rather subtle when compared by single images and by the naked eye. However, when these single images were arranged serially, the differences between the two groups became more apparent and the initial hump, the initial dip and the pseudonormalization time could be assessed even without the indices, after getting used to the visual evaluation. In the E group, the DWI signal intensities increased from Day3 to Day7 in most cases (Figure 1 upper, 1st row) and the change was confirmed to be the initial hump by the rDWI. However, in the EH group, the increase was significantly less and in some cases, no increase was seen at all (absence of the initial hump, Figure 1 lower, 1st row). In addition, in the E group, the increase lasted longer than 9 days, which was regarded as the lack of shortening of the pseudonormalization time (Figure 1 upper, 2nd row) and this was also confirmed by indices. In the EH group, however, the increase returned to a normal level by Day 9 in many cases (the shortened pseudonormalization time, Figure 1 lower, 1st and 2nd row).

The ADC images when observed in a serial manner also showed substantial differences between the E group and the EH group. The degree of reduction of the ADC signal intensities at the lesion sites was less in EH group (Figure 1 lower, 3rd and 4th row) and then, increased to the normal level within Day9, which qualified for the shortening of the pseudonormalization time. On the contrary, in the E group, the ADC image at the lesion site was darker and lasted longer without returning to a normal level within 9 days (lack of shortening of the pseudonormalization time, Figure 1 upper, 3rd and 4th row). The dark ADC intensity at the lesion site became greyish in colour after 9 days in the EH group and the whiteness gradually increased further (late hike) afterwards. In many lesions where the differences were not obvious by the naked eye, the evaluation by the indices still demonstrated significant differences. For an example, in the upper brain stem lesion of the E group (Figure 1 upper, 1st row), the initial hump was not too obvious by the naked eye but the indices (rDWI) were above the normal level of 1.20 on Day3 and Day5 (1.54 and 1.30, respectively), indicating the presence of the initial hump. Since ADC images are more difficult to evaluate by the naked eye, the lack of the pseudonormalization of the lesions such as in the Figure 1 upper, 3rd and 4th row could only be evaluated by the indices (rADC), which, at these lesions, had changed from 0.48 to 0.31 to 065 (3rd row) and 0.79 to 0.39 to 0.82 (4th row) on Day1, Day3 and Day6, respectively. All of these indices were below the normal level of 0.9 and remained depressed longer than Day10 and therefore the changes were regarded as showing the lack of the pseudonormalization (or failure of shortening of the pseudonormalization time). On the other hand, the presence of shortened pseudonormalization time in the EH group was shown by the both indices as in Figure 1 lower lesions. The lesions showed the initial hump of rDWI (2nd row, 2.03) and the initial dip of rADC (4th row, 0.54) on Day2 but these data improved to 1.14 (rDWI, as compared to the normal value of less than 1.2) and to 2.50 (rADC, as compared to the normal value of more than 0.9), by the Day9 (therefore, the shortened pseudonormalization time and late hike).

### Serial rDWI averages in the E group (treated with Edaravone only) and in the EH group (treated with a combination of Edaravone and hydrogen (Figure 2 upper)

**Figure 2:**
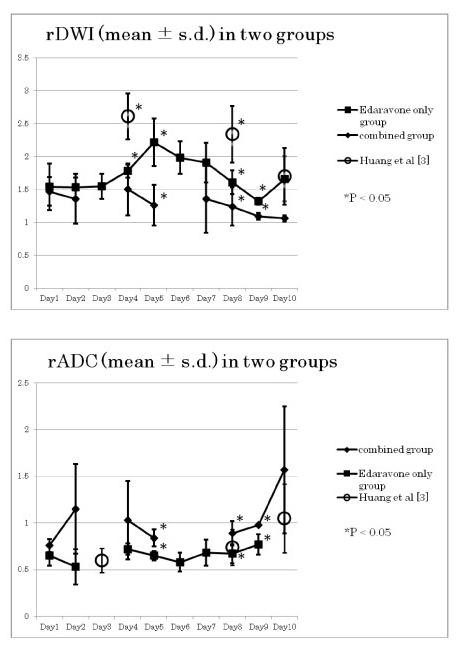
**Serial changes in rDWI (upper) and rADC (lower)**. **upper: **Daily averages of rDWI in the E group patients showed a mild initial hump (Day4 to Day8, up to 2.2) but remained less than a natural course (rDWI of 2.5, Huang et al [3]). In the EH group, the initial hump was not seen (p < 0.05 on the Day 5, 8 and 9). No shortening of the pseudonormalization time was seen in E group (the rDWI average remained above 1.2 by Day9). In the EH group, the rDWI averages on Day 8 reached the normal level of 1.2 (shortened pseudonormalization time). **Lower: **Daily averages of rADCs in the E group patients showed a mild initial dip (Day4 to 7). In the EH group, the initial dip was rather short lived on Day 5 but no data available on Day6 & 7. No pseudonormalization of the rADC was noted within 9 days in the E group. In the EH group, however, the shortening was seen on Day 9. Then, the rADC of EH group increased (late hike). The differences of the rADC in the two groups reached a statistical significance on the Day5, 8 and 9.

Daily averages of rDWI in the E group patients showed a definite initial hump (above 1.2) between Day4 and Day8. However, the highest rDWI averages of the E group remained at 2.1 levels and did not deteriorate as high as 2.5, as in the natural course [3] and the difference was statistically significant on Day4 (Figure 2 upper). On the other hand, the initial hump was not seen in the EH group and the difference was significant (p < 0.05) on the Day5, 8 and 9 (absence of the initial hump). The rDWI averages of the E group did not fall below a normal level of 1.2 by Day10 and thus failed to shorten the pseudonormalization time. However, in the EH group, the rDWI averages on Day8 and Day9 reached 1.2 or less and thus qualified for the shortening of the pseudonormalization time. These findings indicate that the treatment in the E group did not abolish the initial hump and did not shorten the pseudonormalization. However, both conditions were accomplished in the EH group and in this sense, although the differences may appear rather minuscule, the results of the treatment in EH group was superior to those of the E group, when evaluated by the rDWI. The degree of the initial hump of the E group was significantly less and better than that of the natural course, however.

### Serial rADC averages in the E group (treated with Edaravone only) and in the EH group (treated with a combination of Edaravone and hydrogen) (Figure 2 lower)

Daily averages of rADCs in the E group patients showed the initial dip on the Day4 and Day5. In the EH group, however, the initial dip appeared to be delayed and rather short lived on the Day5 and possibly on the Day6 or Day7 but no data available during this period. These patients were usually scheduled for MRA (MRI angiogram) of the cervical carotid artery on the Day3 and other cardiopulmonary studies on Day6 or Day7 and the lack of the MRI data on these hospital days made it difficult to assert the duration of the short lived initial dip. Definite pseudonormalization of the rADC was not noted within 10 days in the E group while in the EH group, the shortening of the pseudonormalization time was seen on Day9. The rADC of the EH group increased gradually afterwards (late hike). The difference of the daily averages between the E group and the EH group reached a statistical significance on the Day5, 8 and 9. The results of the treatment in EH group were, therefore, superior to those of E group when evaluated by the rADC also.

### Neurological outcomes in the E group (treated with Edaravone only) and in the EH group (treated with a combination of Edaravone and hydrogen)

The neurological conditions of the patients recorded on the Day1 and at the time of discharge from the hospital were compared. There were 4, 2 and 20 patients, who were regarded as improved, worse and unchanged, respectively, in the E group. However, all of the patients in the EH group were regarded as unchanged, except one patient who had a very high blood sugar from uncontrolled diabetes and got worse. The neurological evaluation was based upon NIHSS and if the score did not show any change, then, the result of the MMT was added. The difference of the neurological changes in the two groups was statistically not significant.

## Discussion

### MRI analysis

Since MRI scan is an essential part of the diagnosis of the cerebral infarction patients, the effects of the infarction treatment have frequently been evaluated by the MRI scan also. Previous publications utilized the area of DWI abnormality as an equivalent to the size of infarction. However, it is now well known that areas of the DWI abnormality are consisted of heterogeneous tissues and all of the area of DWI abnormalities may not progress to infarction. The increase in the size and density of the DWI abnormality may not reflect worsening and/or expansion of the infarction because the DWI data include T2 sequence of the MRI. Therefore, the increase may simply reflect the increase in water content of the area from vasogenic edema or from proliferated primitive and leaky neovasculature and the phenomena are inclusively called "T2 shine through" [13]. Therefore, if the effects of the treatment were analyzed only by the increase or decrease of the size and density of the DWI abnormality, the analysis may falsely conclude the treatment to be ineffective or effective, respectively. The ADC is not influenced by the T2 change and more valuable than DWI. However, since the ischemic tissue abnormality reduces the ADC data and this makes the area of the ADC abnormality very difficult to discern from the surrounding tissue. Therefore, the analysis of the effects of the treatment based upon the size of the DWI/ADC abnormality was thought to be inappropriate and we adopted the current technique. The technique is to calculate the average number of DWI/ADC raw data within the identical area of the brain within the recorded pixel size in all the MRI images obtained during the hospitalization by using a specific template made for each patient. This appeared to have accomplished the calculation in exactly same area of the same size in a consistent manner. This technique has been utilized in pharmacological evaluation of medications in the ischemic brain in the past but mainly in the animal experiments, probably due to difficulty in obtaining frequent MRI scans in clinical settings.

Our study included only brainstem infarction cases because of ease of defining the perimeter of the lesion for the calculation. The brainstem infarctions are usually round or oval in shape and small and very discrete from the surrounding tissue. In addition, the tissue is mainly consisted of white matter and devoid of CSF space. The MRI indices are influenced by the heterogeneity of the tissue [4] and particularly by the presence of CSF space in the tissue as in the cerebral cortical lesions.

### Neurological evaluation of brainstem infarction patients with NIHSS

All of the patients in the EH group were regarded as neurologically unchanged except one patient after the combined treatment with Edaravone and hydrogen, based upon the NIHSS. However, all of these patients in the EH group except one were satisfied with significant improvement of their preadmission symptoms by the time of discharge from the hospital. NIHSS is the most reliable and most accepted neurological scoring system for stroke patients which is calculated and recorded after performing well described and rather simple neurological examinations. However, these examinations are heavily weighted for the evaluation of anterior circulation stroke. Major symptoms of our brainstem stroke patients were due to posterior circulation abnormality and included dizzy sensation, vertigo without nystagmus, vague and subjective paresthesia of one side of the body with normal touch sensation, difficulty in walking from some swaying and staggering sensation but with normal knee to heel tests, normal diadochokinesis and normal muscle strength, in addition to some sensation of swallowing difficulty with normal gag reflex etc. None of these symptoms are calculable by NIHSS and therefore, the patient's satisfaction in the EH group was not reflected as improvement in the NIHSS.

### Effects of hydroxyl radical scavengers, Edaravone and hydrogen on cerebral infarction

The beneficial effects of Edaravone in the treatment of cerebral infarction have been well established [14]. Edaravone is known for its unique property with both water and lipid solubility and has potent scavenger action against hydroxyl and peroxynitrite radicals and ROS [15]. It acts also in reducing the brain edema of the ischemic brain tissue by protecting endothelial cells from ROS and by keeping integrity of the blood brain barrier and also by reducing the inflammatory responses in the ischemic area of the brain [16]. Initially, Edaravone was thought to be a simple quencher of the radicals but later many neuroprotective properties were found [17,18], and effectiveness in many organs and many disease conditions are added [19,20]. Currently, it is recognized as a most effective scavenger of radicals and also neuroprotective agents in Japanese neurosurgical community but additional clinical studies were discussed in the U.S.A [21].

Hydrogen is also known as a potent scavenger of the hydroxyl and peroxynitrite radicals and does not affect NO production which is advantageous to the ischemic brain tissue. The investigational and clinical interests have been promulgated recently by epochal articles [11] and a review [22]. Direct actions of hydrogen on extracellular and intracellular hydroxyl radical provide protection of mitochondria and nuclear DNA but hydrogen does not harm other cellular elements which relate to signal transduction. When hydrogen was given during reperfusion in an animal ischemic brain model, it protected ischemia-reperfusion injury of the brain, although only when hydrogen was given during the reperfusion but not during the ischemic period. However, these effects were actually better than those of Edaravone and FK506 combination [11]. Since FK506 alone is known to decrease the ischemic brain size, it is remarkable that hydrogen superseded the effects of the combination. In addition, hydrogen demonstrated extended effectiveness in many other organs and in various situations such as in diabetes[23], intestinal grafts[24], tumor growth inhibition [25], allograft nephropathy[26], cardiac ischemia/reperfusion[27], sepsis [28], liver injury [29], haemodialysis[30], spinal cord injury[31], an animal model of Parkinson's disease[32] and Alzheimer's disease[33], in addition to health promotion [34]. Therefore, there is nothing to indicate that hydrogen is inferior to Edaravone for the treatment of cerebral infarction and it is quite possible that a single use of hydrogen is as effective as Edaravone treatment and probably much safer. However, it would be an unethical conduct until larger controlled clinical studies accumulate more evidences, because of limitations of our study. However, if the advantages in the EH group of current study were substantiated in the future studies, the advantages may be due to the increased frequency of administration of the radical scavengers as was in EH group (4 times per day vs. 2 times per day), and/or direct hydrogen effects on the inflammatory cells, chemokines and growth and antiapoptoic factors and/or a direct neutralizing action on the residual radical substances of intermediate Edaravone metabolites in ischemic and hypoxic brain tissue. Edaravone putatively provides electrons and becomes a radical by itself until it reacts with oxygen and then changes, through Edaravone peroxyl radical, to a non-radical material, 2-oxo-3-(phenylhydrazono)-butanoic acid (OPB) [35] which may accumulate in the brain eventually. Hydrogen may have interacted with those intermediate radical products favourably and provided better MRI changes in our study. At the beginning of this study, our concerns included the government approved and recommended Edaravone dose (60 mg/day for 2 weeks = 840 mg) and subsequent blood level dynamics. It is interesting that a currently on going Phase 2 study in Europe increased the Edaravone doses from 840 mg to 1000 mg and 2000 mg [21]. The results of the study may solve some of our concerns.

The limitations of our study include a non-controlled way of patient selection, inclusion of rather small number of the patients particularly in the combined group, use of current NIHSS for neurological evaluation for the brainstem infarction, lack of long term follow-up etc. We are organizing a new study to improve these limitations currently.

## Conclusions

Administration of hydroxyl radical scavengers in acute stage of brainstem infarction improved MRI indices (rDWI, rADC) against the natural course. The favourable effects were more obvious and significant in the EH group (a combined group of Edaravone and hydrogen) as compared to the E group (Edaravone alone group). These findings may imply the need for more frequent daily administration of hydroxyl radical scavenger, or possible presence of additional hydrogen effects on scavenger mechanisms.

## Competing interests

The authors declare that they have no competing interests and were not compensated at all by any pharmaceutical and biotechnology company or any other companies to contribute this article to the peer-reviewed scientific literature.

## Authors' contributions

The authors equally contributed to the production of this article and have read and approved the final manuscript.

## References

[B1] Yang Q Brian M Tress BM Barber PA Desmond PM Darby DG Gerraty RP Li T Davis SM Study of apparent diffusion coefficient and anisotropy in patients with Acute Stroke Stroke 1999 30 2382 2390 10.1161/01.STR.30.11.2382 10548675

[B2] Schwamm LH Koroshetz WJ Sorensen GA Wang B Copen WA Rordorf G Buonanno FS Schaefer PW Gonzalez GR Serial Diffusion-and Hemodynamic-Weighted Magnetic Resonance Imaging Stroke 1998 29 2268 2276 10.1161/01.STR.29.11.2268 9804633

[B3] Huang IJ Chen CY Chung HW Chang DC Lee CC Chin SC Liou M Time Course of Cerebral Infarction in the Middle Cerebral Arterial Territory: Deep Watershed versus Territorial Subtypes on Diffusion-weighted MR Images Radiology 2001 221 35 42 10.1148/radiol.2211001412 11568318

[B4] Fiebach JB Schellinger JO Sartor HW Serial analysis of the apparent diffusion coefficient time course in human stroke Neuroradiology 2002 44 294 298 10.1007/s00234-001-0720-8 11914803

[B5] Liu S Karonen JO Liu Y Vanninen Ft Partanen K Cinbnen MK Vainio P Aronen HJ Serial Measurements of the Apparent Diffusion Coefficient in Human Stroke on Five Time Points over Three Months Proc Intl Sot Mag Reson Med 2000 8 1203

[B6] Huang L Wong XH Li G The application of DWI and ADC map in cerebral infarction Proc Intl Soc Mag Reson Med 2001 9 1446

[B7] Marks MP Tong DC Beaulieu C Albers GW deCrespigny A Moseley MR Evaluation of early reperfusion and i.v. tPA therapy using diffusion-and perfusion-weighted MRI Neurology 1999 52 1792 1798 10371525 10.1212/wnl.52.9.1792

[B8] Schaefer POW Hassankhani A Putman C Sorensen GA Schwamm L Koroshez W Gonzalez GR Characterization and evolution of diffusion MNRE imaging abnormalities in Stroke patients undergoing intra-arterial thrombolysis ANJR 2004 25 951 957 PMC7975652 15205129

[B9] Tanaka M Pharmacological and clinical profile of the free radical scavenger edaravone as a neuroprotective agent Folia Pharmacol Jpn 2002 119 301 308 10.1254/fpj.119.301 12061142

[B10] Kageyama M Toriyama S Tsubosita A Muraki S Yamada T Ishibashi A A post-marketing drug use survey of a neuroprotecive drug Radicut injection 30 mg(non-proprietary name: edaravone) for acute ischemic stroke J New Rem Clin 2009 58 1212 1226

[B11] Ohsawa I Ishikawa M Takahashi K Watanabe M Nishimaki K Yamagata K Katsura K Katayama Y Asoh S Ohta S Hydrogen acts as a therapeutic antioxidant by selectively reducing cytotoxic oxygen radicals Nat Med 2007 13 688 694 10.1038/nm1577 17486089

[B12] Takagi M Concept of branch atheromatous disease Neurol Med 2008 69 542 549

[B13] Burdett JH Elster AD Ricci PE Acute Cerebral Infarction: Quantification of Spin-Density and T2 Shine-through Phenomena on Diffusion-weighted MR Images Radiology 1999 212 333 339 10429687 10.1148/radiology.212.2.r99au36333

[B14] Edaravone Acute Infarction Study Group Effect of a novel free radical scavenger, edaravone (MCI-186), on acute brain infarction. Randomized, placebo-controlled, double-blind study at multicenters Cerebrovasc Dis 2003 15 222 229 12715790 10.1159/000069318

[B15] Yamamoto Y Kuwahara T Watanabe K Watanabe K Antioxidant activity of 3-methyl-1-phenyl-2-pyrazolin-5-one Redox Report 1996 2 333 338 10.1080/13510002.1996.11747069 27406414

[B16] Zhang N Komine-Kobayashi M Tanaka R Liu M Mizuno Y Urabe T Edaravone reduces early accumulation of oxidative products and sequential inflammatory responses after transient focal ischemia in mice brain Stroke 2005 36 2220 2225 10.1161/01.STR.0000182241.07096.06 16166574

[B17] Kawai H Nakai H Suga M Yuki S Watanabe T Saito KI Effects of a novel free radical scavenger, MCl-186, on ischemic brain damage in the rat distal middle cerebral artery occlusion model J Pharmacol Exp Ther 1997 281 921 927 9152402

[B18] Yoshida H Metoki N Ishikawa A Imaizumi T Matsumiya T Tanji K Ota K Ohyama C Satoh K Edaravone improves the expression of nerve growth factor in human astrocytes subjected to hypoxia/reoxygenation Neurosci Res 2010 66 284 289 10.1016/j.neures.2009.11.011 19954754

[B19] Zhang W Sato K Hayashi T Omori N Nagano I Kato S Horiuchi S Abe K Extension of ischemic therapeutic time window by a free radical scavenger, Edaravone, reperfused with tPA in rat brain Neurol Res 2004 26 342 348 10.1179/016164104225014058 15142331

[B20] Watanabe T Tahara M Todo S The Novel Antioxidant Edaravone: From Bench to Bedside Cardiovascular Therapeutics 2008 26 101 114 10.1111/j.1527-3466.2008.00041.x 18485133

[B21] Lapchak PA A critical assessment of edaravone acute ischemic stroke efficacy trials: is edaravone an effective neuroprotective therapy? Expert Opinion on Pharmacotherapy 2010 11 1753 1763 10.1517/14656566.2010.493558 20491547 PMC2891515

[B22] Nakao A Sugimoto R Billiar TR McCurry KR Therapeutic antioxidant medical gas J Clin Biochem Nutr 2009 44 1 13 10.3164/jcbn.08-193R 19177183 PMC2613492

[B23] Kajiyama S Hasegawa G Asano M Hosoda H Fukui M Nsksmura N Kitawaki J Imai S Nakano K Ohta M Adachi T Obayashi H Yoshikawa T Supplementation of hydrogen-rich water improves lipid andd glucose metabolism in patients with type 2 diabetes or impaired glucose tolerance Nur Res 2008 28 137 143 10.1016/j.nutres.2008.01.008 19083400

[B24] Buchholz BM Kaczorowski DJ Sugimoto R Yang R Wang Y Billiar TR McCurry KR Bauer AJ Nakao A Hydrogen inhalation ameliorates oxidative stress in transplantation induced intestinal graft injury Am I transplant 2008 8 2015 2024 10.1111/j.1600-6143.2008.02359.x 18727697

[B25] Saitoh Y Okayasu H Xiao L Harata Y Miwa N Neutral pH hydrogen-enriched electrolyzed water achieves tumor-preferential clonal growth inhibition over normal cells and tumor invasion inhibition concurrently with intracellular oxidant repression Oncol Res 2008 17 247 255 10.3727/096504008786991620 19192719

[B26] Cardinal JS Zhan J Wang Y Sugimoto R Tsung A McCurry KR Billar R Nakao A Oral administration of hydrogen water prevents chronic allograft nephropathy in rat renal transplantation Kidney Int 2010 77 101 109 10.1038/ki.2009.421 19907413

[B27] Nakao A Kaczorowski DJ Wang Y Cardinal JS Buchholz BM Sugimoto R Tobita K Lee S Toyoda Y Billar TR McCurry KR Amelioration of rat cardiac cold ischemia/reperfusion injury with inhaled hydrogen or carbon monoxide, or both J Heart Lung Transplant 2010 29 544 553 10.1016/j.healun.2009.10.011 20036162

[B28] Xie K Yu Y Pei Y Hou L Chen S Xiong L Wang G Protective effects of hydrogen gas on murine polymicrobial sepsis via reducing oxidative stress and HMGB1 release Shock 2010 34 90 97 19997046 10.1097/SHK.0b013e3181cdc4ae

[B29] Liu Q Shen WF Sun HY Fan DF Nakao A Cai JM Yan G Zhou WP Shen RX Yang JM Sun XJ Hydrogen-rich saline protects against liver injury in rats with obstructive jaundice Liver Int 2010 30 958 968 10.1111/j.1478-3231.2010.02254.x 20492513

[B30] Nakayama M Nakao H Hamada H Itami N Nakazawa R Ito S A novel bioactive hemodialysis system using dissolved dihydrogen(H2) produced by water electrolysis: a clinical trial Nephrol Dial Transplant 2010 25 3026 3033 10.1093/ndt/gfq196 20388631

[B31] Chen CW Chen QB Mao YF Xu SM Xia CY Shi XY Zang ZH Yuan HB Sun XJ Hydrogen-rich saline protects against spinal cord injury in rats Neurochem Res 2010 35 1111 1118 10.1007/s11064-010-0162-y 20354783

[B32] Fujita K Seike T Yutsudo N Ohno M Yamada H Yamaguchi H Sakumi K Yamakawa Y Kido M Takaki A Katafuchi T Tanaka Y Nakabeppu y Noda M Hydrogen in drinking water reduces dopaminergic neuronal loss in the 1-methyl-4-phenyl-1,2,3,6-tetrahydropyridine mouse model of Parkinson disease PloS One 2009 30 1 10 e7247 10.1371/journal.pone.0007247 PMC2747267 19789628

[B33] Li J Wang C Zhang JH Cai JM Cao YP Sun XJ Hydrogen-rich saline improves memory function in a rat model of amyloid-beta-induced Alzheimer's disease by reduction of oxidative stress Brain Res 2010 1328 152 161 20171955 10.1016/j.brainres.2010.02.046

[B34] Nakao A Toyoda Y Sharma P Evans M Guthrie N Effectiveness of Hydrogen Rich Water on Antioxidant Status of Subjects with Potential Metabolic Syndrome--An Open Label Pilot Study J Clin Biochem Nutr 2010 46 140 149 10.3164/jcbn.09-100 20216947 PMC2831093

[B35] Higashi Y Jitsuiki D Chayama K Yoshizumi M Edaravone (3-methyl-1-phenyl-2-pyrazolin-5-one), a novel free radical scavenger, for treatment of cardiovascular disease Recent Patents on Cardiovascular Drug Discovery 2006 1 85 93 10.2174/157489006775244191 18221078

